# Supplementary material for: Systematic Review of Zinc’s Benefits and Biological Effects on Oral Health
Source: Materials (Basel). 2024 Feb 7;17(4):800. doi: 10.3390/ma17040800 (PMC10890596; doi:10.3390/ma17040800)
Supplement: Supplementary file 1 [file Table S1.pdf › Table S1.pdf]

	Item 1	Item 2	Item 3	Item 4	Item 5	Item 6	Item 7	Item 8	Item 9	Item 10	Item 11	Item 12	Item 13	Final Score
**IN VIVO STUDIES**														
**Doneria 2017**	3	3	3	3	2	3	2	3	2	2	3	N/A	3	32/36
**Hagenfeld 2019**	3	3	2	3	2	3	3	3	3	3	3	2	2	35/39
**Moura 2021**	3	3	3	3	3	3	3	3	3	3	3	N/A	3	36/36
**Prasad 2018**	3	3	3	3	3	3	3	3	3	3	3	2	2	37/39
**Sreenivasan 2020**	3	2	3	2	3	3	3	3	2	3	3	2	2	34/39
**IN VITRO STUDIES**														
**CONSERVATIVE AND CARIOLOGY**														
**Barma 2021**	3	3	3	3	3	3	3	3	3	3	3	N/A	2	35/36
**Eskandarizadeh 2019**	3	3	2	3	3	3	3	2	3	3	3	N/A	2	33/36
**Garcia 2021**	3	2	3	3	3	3	3	3	2	3	3	N/A	3	34/36
**Huang 2020**	3	3	3	2	2	3	2	3	3	2	3	3	3	36/39
**Lavaee 2018**	3	3	3	3	3	3	3	3	2	3	3	2	3	37/39
**Manus 2018**	3	3	3	3	3	3	3	2	3	3	3	N/A	3	35/36
**Mirhosseini 2019**	3	3	3	2	3	3	2	3	2	3	3	N/A	3	33/36
**Peralta 2018**	3	3	3	3	3	3	3	3	3	3	3	3	2	38/39
**Steiger 2020**	3	2	3	3	3	3	2	3	3	3	3	N/A	3	34/36
**Suzuki 2018**	3	3	2	3	2	2	3	3	3	3	3	3	3	35/39
**Tabatabaei 2019**	3	3	3	3	3	3	3	3	3	3	3	2	3	38/39
**Toledano-Osorio 2018**	3	3	3	3	3	3	3	3	3	3	3	N/A	3	36/36
**Xu 2020**	3	3	2	3	3	3	2	3	3	3	3	2	3	36/39
**ENDODONTICS**														
**Fan 2018**	3	2	3	3	2	3	2	3	3	3	3	2	2	34/39
**Garcia 2018**	3	3	3	2	3	3	2	2	3	3	3	3	2	35/39
**Garcia 2020**	3	3	3	3	2	3	3	3	2	3	3	3	2	36/39
**Pilownic 2017**	3	2	3	3	2	3	3	2	3	3	2	N/A	3	32/36
**PERIODONTOLOGY AND IMPLANTOLOGY**														
**Chen 2021**	3	3	3	3	2	3	2	3	2	2	3	N/A	3	32/36
**Fröber 2020**	3	3	2	3	3	3	3	2	3	3	3	N/A	2	33/36
**Lin 2021**	3	2	3	3	3	3	2	3	3	2	2	N/A	3	32/36
**Sánchez 2018**	3	2	3	3	3	3	2	3	3	3	3	N/A	3	34/36
**Vergara-Llanos 2020**	3	3	3	3	3	3	3	3	3	3	3	3	2	38/39
**ORTHODONTICS**														
**Kachoei 2021**	3	3	3	3	3	3	3	3	2	3	3	2	3	37/39
**Kim 2018**	3	3	3	3	3	3	3	3	3	3	3	3	2	38/39
**Zeidan 2022**	3	3	3	3	3	3	3	2	3	3	3	3	2	37/39
**OTHER ASPECTS**														
**Chen 2021b**	3	3	3	3	3	3	3	3	3	3	3	N/A	3	36/36
**Mishra 2020**	3	2	3	3	3	3	2	3	3	2	2	N/A	3	32/36
**Wiesmann 2021**	3	3	2	3	2	2	3	3	3	3	3	3	3	35/39
